# Droplets sitting on thin elastic sheets: a study with the boundary element method

**DOI:** 10.1140/epje/s10189-026-00620-9

**Published:** 2026-09-28

**Authors:** Salik Sultan, Josua Grawitter, Gonçalo C. Antunes, Holger Stark

**Affiliations:** Division of Theoretical Physics, Institute of Physics and Astronomy, Technische Universität Berlin, Hardenbergstr. 36, 10623 Berlin, Germany

## Abstract

**Abstract:**

Elasto-capillarity of a droplet wetting an elastic sheet provides an interesting system, both for fundamental and applied research. The droplet sinks into the sheet and assumes the shape of a lens. To determine the equilibrium shape in simulations, we formulate a boundary element method (BEM) extending our earlier approaches, and apply the BEM to three specific protocols for the boundary conditions of the sheet. For a clamped elastic sheet, we use various morphological metrics to demonstrate that the lens shape crucially depends on the sheet thickness. Stretching the sheet isotropically allows for an additional control parameter to influence the droplet shape and the tension in the sheet, which we quantify by radial profiles of the azimuthal and radial elastic stresses. We further demonstrate how the focal length of a liquid lens can be tuned by varying the applied tension. Finally, stretching the sheet along one direction elongates the droplet, and the sheet shows folds and dimples.

**Graphical abstract:**

A droplet wetting a clamped elastic sheet assumes a lens shape (bottom); stretching the sheet along one direction, the droplet also elongates and the sheet shows folds and dimples (top)

**Figure:**
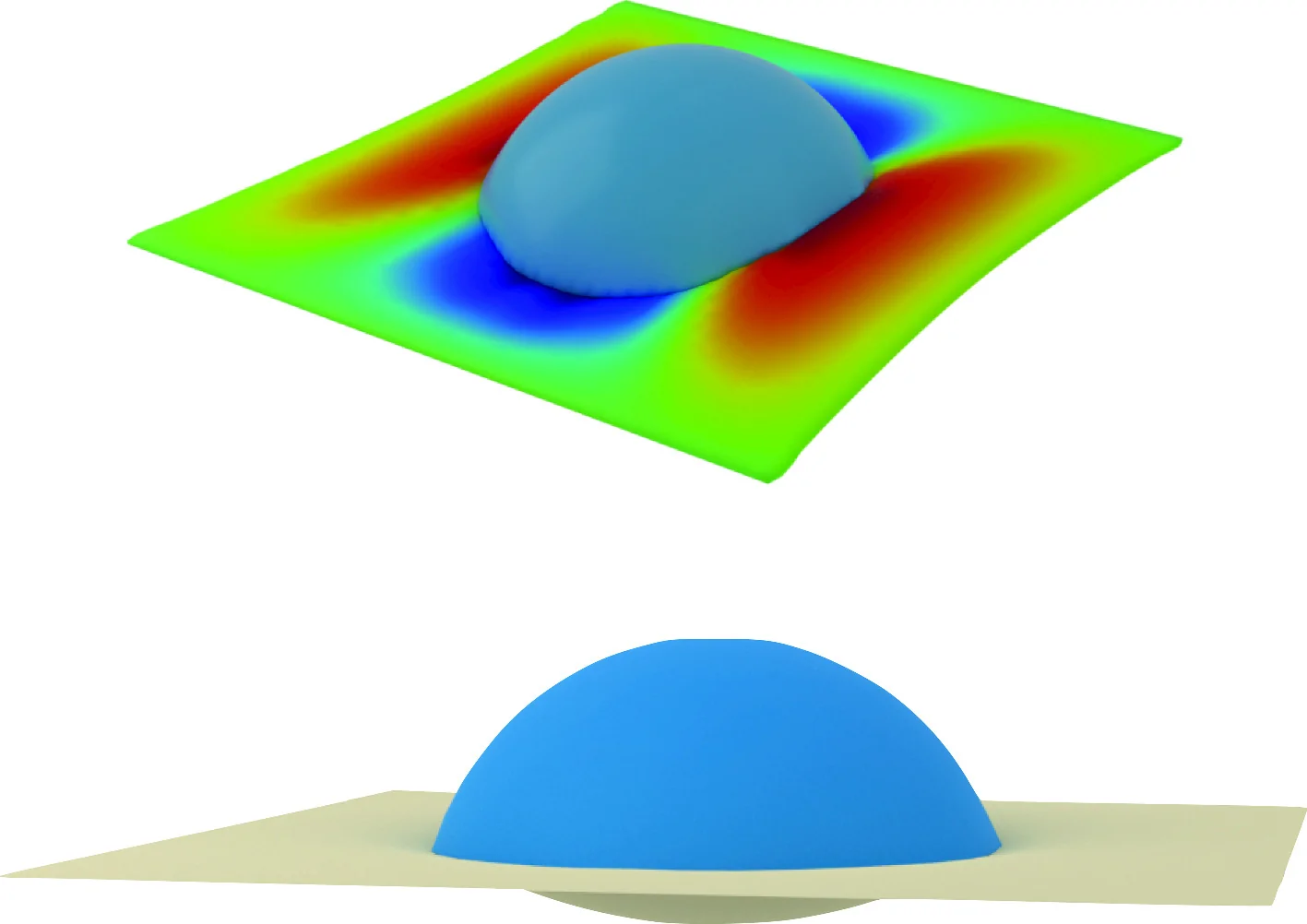

**Supplementary Information:**

The online version contains supplementary material available at https://doi.org/10.1140/epje/s10189-026-00620-9.

## Introduction

Many biological and synthetic solids, including tissue, plastics, slender rods, and fibrous media are mechanically flexible and respond differently to wetting by a liquid than rigid substrates [1, 2]. This phenomenon is termed elasto-capillarity. The flexibility of the substrates can vary depending on the material and environmental conditions. Furthermore, the direction-dependent flexibility of anisotropic materials gives rise to additional complexity, also regarding the statics and dynamics of the contact line [3]. A particularly interesting property is the Shuttleworth effect, where the surface stress depends on the induced strain [4–6].

Thus, the competition of capillary and elastic stresses has been subject of intense research efforts in recent years [1, 2, 5–7]. Particular emphasis was given to the region around the contact line, which was visualized in experiments [8–10]. Furthermore, the equivalence of the microscopic contact angle with Young’s angle for droplets sitting on elastic solids [11, 12] and sheets [13–15] has been investigated for several decades.

Droplets also show durotaxis, where they move toward softer regions of the substrate [16], in contrast to living tissues [17], and even perform stick–slip motion [10]. Experiments with synthetic hydrogels demonstrate that the stiffness of the substrate can be tuned by light [18]. In theory, a unified numerical model for a droplet moving on a soft solid has been proposed recently [19], a gradient-dynamics model that treats several droplets has been formulated [20], and a thin-film model reproduced the durotaxis of a droplet [21]. Finally, wetting transitions on soft substrates were studied [22].

A particularly interesting realization of a soft substrate are thin elastic sheets. Early experiments demonstrated appealing wrinkling patterns around sessile droplets [23–25]. This observation sparked further experiments with highly deformable elastomeric sheets [26], where the droplet shape becomes anisotropic under uniaxial stress [27, 28]. Droplets covered by an elastic sheet show atypical equilibrium shapes [29], they can become completely wrapped after a high-speed impact on the sheet [30], and show capillary origami [31]. Experiments with thin sheets under tension have been reviewed in Ref. [32] and detailed theoretical studies exist [13, 14, 32–34], including an investigation of the motion of thin drops on an elastic sheet [35]. Finally, we point to nanoporous silicon as a novel material, which can be used to fabricate actuatable elastic sheets [36, 37].

Beyond exhibiting interesting phenomenology, elastocapillary effects are opening new technological avenues. For example, a droplet sitting on an elastic sheet deforms the sheet and assumes the shape of a lens [32]. Micro-optofluidic lenses have been an attractive development since their shape and thereby their properties can easily be tuned by electric fields [38–40], pressure used in commercially available lenses [41], as well as an applied tension, as we demonstrate explicitly in this article. Furthermore, a very topical field currently are biocondensates. They are present in the cell and wet the cell membrane [42–44]. This system has recently been modeled by a phase-field approach [45].

So far, we have considered flexible substrates that influence (dynamic) wetting and, in particular, the droplet shape. From a broader perspective, droplet shape and dynamic wetting can be controlled by varying the wettability of a substrate. This can be done through spatio-temporal patterns of the equilibrium contact angle, as in the seminal works of Refs. [46, 47], or by modulating the substrate height and thereby inducing curvi-propulsion [48], which also gives rise to the inverted Cheerios effect [49]. In fact, the numerical method, which we will extend in this article, was developed to describe dynamic wetting due to wettability patterns and curved substrates [50–52]. In particular, light in combination with light-switchable substrate molecules [47, 53–56] or micropillars [57, 58] are used to create gradients in wettability, which also serves as an alternative to electrowetting techniques [59]. Finally, wetting of elastic substrates is a special form of adaptive wetting. While the contact with the droplet deforms a flexible substrate, also the surface properties of the substrate can adapt to the presence of a liquid [60, 61]. A broad view on the topic of adaptive wetting is presented in a roadmap article attached to the topical issue *Dynamic Wetting on Adaptive, Flexible and Switchable Substrates*, where also this article is published [62].

In this article we formulate a boundary element method (BEM) to simulate droplets sitting on an elastic sheet. The method follows our earlier approaches, where we studied droplets sitting on spatio-temporal wettability patterns and on undulating substrates [50–52]. After extending the formalized BEM of Ref. [52] to include elastic deformations of the sheet, we demonstrate its applicability. The lens shape of the droplet sitting on a clamped elastic sheet crucially depends on the sheet thickness, which determines the in-plane elastic and bending moduli of the sheet. We also demonstrate that the microscopic contact angle, revealed by a close-up of the region around the contact line, agrees with Young’s angle. However, it differs from a macroscopic contact angle determined by the overall shapes of the two halves of the droplet that make up the lens shape. Stretching the sheet isotropically, allows to control the tension in the sheet, which we quantify by radial profiles of the azimuthal and radial elastic stresses. Furthermore, we argue that changing the lens shape under tension, provides a means to tune the focal length of liquid optical lenses. Finally, when stretching the sheet uniaxially, the droplet elongates and the sheet shows folds and dimples. They are strongest for free lateral edges of the sheet.

The article is organized as follows. We begin by introducing our extended boundary element method in Sect. 2, report the results of the three specific protocols in Sect. 3, and summarize as well as conclude in Sect. 4.

##  Extended boundary element method

A droplet sitting on a thin elastic sheet deforms the latter due to the Laplace pressure that acts within the droplet and pushes against the sheet. In this section, we establish the relevant dynamic equations to describe and simulate the relaxation toward the equilibrium lens shape of the droplet.

Central to the droplet dynamics is the fluid flow within and at the surface of the droplet, where the surface velocity governs changes in droplet shape. Fluid flow at low Reynolds number, as considered in this work, is described by the Stokes equations, $\eta \boldsymbol{\nabla }^2 \boldsymbol{v} -\boldsymbol{\nabla }p=0$, and the incompressibility condition $\boldsymbol{\nabla } \cdot \boldsymbol{v} = 0$. Here, η is the shear viscosity and *p* is pressure. The boundary integral equation solves the Stokes equations by first determining the velocity field at the surface, from which the bulk velocity field can then be deduced [63, 64]. This procedure is the basis of the boundary element method (BEM) [63], which we implemented in Refs. [50, 51] for droplets moving on a substrate with spatio-temporal variations of the wettability.

In a following article, we considered a droplet on an undulating substrate and realized that we needed to reformulate the BEM for the droplet motion in a more formalized way [52]. Essentially, we developed equations for the overdamped dynamics of the droplet surface, where part of the friction matrix is derived from the boundary integral equation and the driving force is the derivative of the droplet free energy. Here, we follow the same route but need to extend this method. In addition to determining the droplet motion, we also need to describe deformations of the elastic sheet induced by the wetting droplet. We call our whole formalism an extended boundary element method since the deformations of the elastic sheet require a separate description.

We describe the extended method in a compact form using continuous field variables and, then, in subsequent sections describe all quantities in more detail. Besides the surface velocity field of the droplet, $\dot{\boldsymbol{R}}_0$, we also introduce the velocity field $\dot{\boldsymbol{R}}_{\text {sh}}$ of the two-dimensional elastic sheet, where $\boldsymbol{R}_0$ and $\boldsymbol{R}_{\text {sh}}$ describe the shape of the droplet surface and elastic sheet, respectively. The dynamic equations become:

1 $$\begin{aligned} \boldsymbol{G} \dot{\boldsymbol{R}} = \left[ \begin{array}{cc} \boldsymbol{G}_0&  \boldsymbol{G}_\text {slip} \\ \boldsymbol{G}_\text {slip}^\text {t}&  \boldsymbol{G}_\text {sh} \end{array} \right] \left[ \begin{array}{c} \dot{\boldsymbol{R}}_{0} \\ \dot{\boldsymbol{R}}_{\text {sh}} \end{array} \right] = - \boldsymbol{\nabla }_{\boldsymbol{R}} \mathcal {F} \,. \end{aligned}$$

Here, $\mathcal {F} = \mathcal {F}_0 + \mathcal {F}_\text {sh} + \mathcal {F}_\text {cons}$ is the total free energy, which consists of the free energies of the droplet ($\mathcal {F}_0$) and the elastic sheet ($\mathcal {F}_\text {sh}$), while $\mathcal {F}_\text {cons}$ contains constraints. They enforce constant droplet volume, with a pressure $p_0$ as Lagrange parameter, and keep part of the elastic sheet always in contact with the droplet. For a continuous field variable $\boldsymbol{R}$, the symbol $\boldsymbol{\nabla }_{\boldsymbol{R}}$ means functional derivative. Thus, the force $-\boldsymbol{\nabla }_{\boldsymbol{R}_0} \mathcal {F}_0$ together with $p_0$ provides the Laplace pressure and Young’s force at the contact line (see Ref. [52]), whereas $-\boldsymbol{\nabla }_{\boldsymbol{R}_\text {sh}} \mathcal {F}_\text {sh}$ contains in-plane elastic shear and dilatation forces as well as bending forces. The friction kernel $\boldsymbol{G}_0$, which in the discretized equations becomes a matrix, is due to viscous shear flow as described by the boundary integral equation and contains contact-line friction, while $\boldsymbol{G}_\text {sh}$ introduces a phenomenological friction coefficient to dampen the sheet motion. It may also be interpreted as, e.g., capturing the effect of an additional fluid phase in contact with the other side of the elastic sheet. Liquid–substrate friction between droplet and elastic sheet is quantified by $\boldsymbol{G}_\text {slip}$ and also contributes to $\boldsymbol{G}_0$, $\boldsymbol{G}_\text {sh}$.

**Fig. 1:**
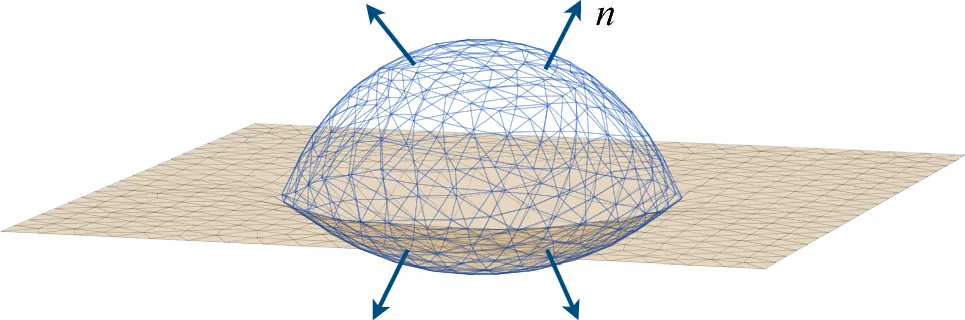
Triangular meshes of gridpoints for the droplet surface [blue, $\boldsymbol{R}_0 = (\boldsymbol{r}_1, \boldsymbol{r}_2, \boldsymbol{r}_3, \ldots )$] and the sheet [brown, $\boldsymbol{R}_\text {sh} = (\boldsymbol{r}_\text {sh1}, \boldsymbol{r}_\text {sh2}, \boldsymbol{r}_\text {sh3}, \ldots )$]. The surface normals $\boldsymbol{n}$ point away from the droplet volume

As illustrated in Fig. 1 we approximate droplet surface and elastic sheet by two triangular meshes so that configuration vector $\boldsymbol{R}_0 = (\boldsymbol{r}_1, \boldsymbol{r}_2, \boldsymbol{r}_3, \ldots )$ contains all vertices of the droplet mesh and $\boldsymbol{R}_\text {sh} = (\boldsymbol{r}_\text {sh1}, \boldsymbol{r}_\text {sh2}, \boldsymbol{r}_\text {sh3}, \ldots )$ all vertices of the sheet mesh. Then, $\boldsymbol{\nabla }_{\boldsymbol{R}}$ becomes the gradient operator with respect to $\boldsymbol{R}$, the force $- \boldsymbol{\nabla }_{\boldsymbol{R}} \mathcal {F}$ a high-dimensional column vector, and $\boldsymbol{G}$ a matrix. Finally, we note that we choose separate meshes for sheet and droplet, also where they are in contact with each other, since the droplet can slip over the sheet and also the contact line moves along the sheet.

In Sects. 2.1– 2.3 we describe all contributions to Eq. (1), also based on Ref. [52], where our approach is fully justified. In Sect. 2.1 we introduce the free energies of droplet and sheet including constraints, while in Sect. 2.2 all contributions to the friction matrix are formulated. In Sect. 2.3 we summarize the final equations, and in Sect. 2.4 we non-dimensionalize them, introduce relevant parameters, and summarize simulation details.

### Droplet-sheet free energy and constraints

First, we collect and explain the different contributions to the free energy $\mathcal {F} = \mathcal {F}_0 + \mathcal {F}_\text {sh} + \mathcal {F}_\text {cons}$.

#### Droplet free energy

The droplet free energy is determined by integrating the surface tension over the surfaces of the droplet and the substrate, which in our case is the elastic sheet. For the first part of the free energy, we rely on the form used in Ref. [52], where we also included a spatio-temporal wettability pattern through the equilibrium contact angle $\theta _\text {eq}(\boldsymbol{r})$. In total, we obtain

2 $$\begin{aligned} \mathcal {F}_0 = \gamma _\text {lg} A_\text {lg} - \gamma _\text {lg} \int \limits _{\mathcal {A}_\text {sl}} \cos \theta _\text {eq}(\boldsymbol{r}) \text {d}^2\boldsymbol{r} + \tau \int _\text {cl} \sin \theta \text {d}r \,. \end{aligned}$$

Here, $\gamma _\text {lg}$ is the surface tension and $A_\text {lg}$ the area of the liquid–gas interface. We also introduce the equilibrium contact angle by using Young’s law, $\gamma _\text {sl}-\gamma _\text {sg} = - \gamma _\text {lg} \cos \theta _\text {eq}$, and the integration domain $\mathcal {A}_\text {sl}$ of the substrate–liquid interface with area $A_\text {sl}$. In what follows, we do not vary the contact angle, so $- \gamma _\text {lg} \cos \theta _\text {eq}$ just represents the difference of the constant surface tensions of the substrate–liquid and the substrate–gas interfaces. Thus, we have

3 $$\begin{aligned} \mathcal {F}_0 = \gamma _\text {lg} A_\text {lg} - \gamma _\text {lg} \cos \theta _\text {eq} A_\text {sl} + \tau \int _\text {cl} \sin \theta \text {d}r \,. \end{aligned}$$

Note that, strictly speaking, this formula neglects changes in the total sheet area during deformation.

To motivate the last term on the right-hand side, where we integrate along the contact line as indicated by the label cl, we note that at the contact line the liquid–gas surface tension pulls up a ridge from a soft substrate with a lateral extension given by the elastocapillary length $l_\text {el} = \gamma _\text {lg} / E$, where *E* is the Young modulus of the sheet material [3, 6]. As we show in Sect. 2.4, for our concrete parameter values, this length is much smaller than the mesh size in our simulations. In such a case, where this deformation is not resolved, one can account for the respective deformation energy by an effective line tension τ. Since the ridge is determined by the component of the tension force pulling perpendicular to the sheet, the factor $\sin \theta$ comes in, where θ is the dynamic contact angle. However, for elastic sheets this line tension can be safely neglected as stated in Ref. [3], since the cross section of the ridge is negligibly small. Hence, we performed all our simulations with τ=0.

#### Constraints

There are two constraints in the droplet-sheet system, which we add to the free energy using Lagrange multipliers. First, the volume of the droplet is constant and, second, the droplet and sheet surfaces are tightly connected to each other at the common interface. Both constraints contribute the free energy

4 $$\begin{aligned} \mathcal {F}_\text {cons} = p_0(V_0-V)+ \int \limits _{\mathcal {A}_\textrm{sl}} \lambda (\boldsymbol{x}) \left[ z - z_\text {sh}(\boldsymbol{x})\right] \text {d}^2 \boldsymbol{r} \,, \end{aligned}$$

where the first term on the right-hand side constrains the droplet volume to the value $V_0$ and the Lagrange multiplier $p_0$ is a pressure. For computational purposes we note that with the help of Gauss’s theorem the droplet volume can be expressed as $V=\frac{1}{3}\oint \boldsymbol{r} \cdot \boldsymbol{n} \text {d}^2\boldsymbol{r}$, where the unit vector $\boldsymbol{n}$ points along the outward normal, and the integration is performed over the whole droplet surface. Furthermore, we use the Monge form to parametrize how the flat elastic sheet in the *x*-*y* plane is deformed by the droplet along the vertical *z* axis using the height function, i.e., the vertical coordinate of the sheet, $z_\text {sh}(\boldsymbol{x})$ with $\boldsymbol{x} = (x,y)$. So, the second term constrains the height *z* of the droplet-sheet interface to the height variations of the sheet using the Lagrange multipliers $\lambda (\boldsymbol{x})$.

For the implementation in a simulation code, it makes sense to write the discretized version of the constraints in Eq. (4) in the compact form

5 $$\begin{aligned} \mathcal {F}_\text {cons} = \sum \limits _{i=0}^N \lambda _i g_i(\boldsymbol{R}) \,, \end{aligned}$$

where we set $\lambda _0 = p_0$ and $g_0 = V_0-V$. Furthermore, the droplet-sheet interface is discretized by *N* gridpoints, and we have for the connectivity constraint of the *i*-th node, $g_i(\boldsymbol{R}) = z_i - z_{\text {sh}i}(x_i, y_i)$, with i=1...N. Here, an area element from discretizing the integral in Eq. (4) is subsumed into $\lambda _i$.

To compute $\mathcal {F}_0$ from Eq. (3) and $\mathcal {F}_\text {cons}$ from Eq. (4) numerically, we formulate them as sums over the mesh triangles, as detailed in Ref. [52]. Then, we calculate the integrals over the triangles either by Gaussian quadrature or using a linear interpolation of the integrand between the gridpoints. This provides the free energies as functions of the gridpoints from droplet and sheet. The gradient $- \boldsymbol{\nabla }_{\boldsymbol{R}} (\mathcal {F}_0 + \mathcal {F}_\text {cons})$ is then computed by automatic differentiation using the Julia program package ForwardDiff.jl.

#### Sheet free energy

The sheet free energy consists of two contributions that describe in-plane shear and dilatation deformations, as well as out-of-plane bending, $\mathcal {F}_\text {sh} = \mathcal {F}_\text {d} + \mathcal {F}_\text {b}$. We have used both free energies in previous work [65, 66].

**Skalak model** To consider in-plane deformations, we rely on the Skalak model developed to address deformations of red-blood cells as one example of a hyperelastic material [67]. We describe any material point of the undeformed plane sheet in the *x*-*y* plane by the two-dimensional vector $\boldsymbol{\xi } = (\xi _1, \xi _2)$, which due to in-plane and bending deformations moves to a point with the three-dimensional position vector $\boldsymbol{r}_\text {sh}$. With the help of the deformation gradient tensor $F_{i\alpha } = \partial r_{\text {sh},i}/ \partial \xi _\alpha$ (i=1,2,3 and α=1,2), we introduce the Cauchy strain tensor $\boldsymbol{C} = \boldsymbol{F}^\text {t} \boldsymbol{F}$ with components $C_{\alpha \beta } = F_{i\alpha } F_{i\beta }$ that completely describes in-plane shear and dilatation deformations. The components $C_{\alpha \alpha }$ and $C_{12}$ quantify, respectively, stretching factors along the α-directions and the sheared angle between directions 1 and 2.

The Skalak model uses invariants of the Cauchy strain tensor, $I_1 = \text {tr} \boldsymbol{C} - 2$ and $I_2 = \text {det} \boldsymbol{C} -1$, to construct an elastic free energy also valid for large deformations,

6 $$\begin{aligned} \mathcal {F}_\text {d}= &   \int _{\mathcal {A}_\text {sh}} f_\text {d} \, \text {d}^2\xi \nonumber \\= &   \int _{\mathcal {A}_\text {sh}} \Big [\frac{\kappa _S}{12} (I_1^2 + 2 I_1 - 2 I_2) + \frac{\kappa _A}{12} I_2^2\Big ] \, \text {d}^2\xi , \end{aligned}$$

where $f_d$ is the surface free energy density, and $\kappa _S$ and $\kappa _A$ are the respective shear and area dilatation moduli. We have also indicated the integration domain of the elastic sheet with $\mathcal {A}_\text {sh}$. Note that the integral is performed over the material coordinates $\xi _1$ and $\xi _2$. Now, when calculating the elastic forces $-\boldsymbol{\nabla }_{\boldsymbol{R}_\text {sh}} \mathcal {F}_\text {d}$ in the discretized form for the dynamic equation (1), we rely on a procedure outlined in Ref. [68]. It calculates the elastic forces analytically as a function of $\boldsymbol{R}_\text {sh}$ by explicitly considering deformations of the single mesh triangles.

For completeness, for small deformations we connect the Skalak model to the theory of linear elasticity, where the Cauchy strain tensor becomes $\boldsymbol{C}= 1 + 2 \boldsymbol{\epsilon }$ and $\boldsymbol{\epsilon }$ is the conventional strain tensor. Then the Skalak free energy can be written in the form,

7 $$\begin{aligned} \mathcal {F}_\text {d} = \int _{\mathcal {A}_\text {sh}} \Big [ \mu _\text {2D} \boldsymbol{\epsilon } \cdot \boldsymbol{\epsilon } + \frac{\lambda _\text {2D}}{2} (\text {tr} \boldsymbol{\epsilon })^2 \Big ] \, \text {d}^2r_\text {sh} \end{aligned}$$

with $\boldsymbol{\epsilon } \cdot \boldsymbol{\epsilon } = \epsilon _{ij}\epsilon _{ij}$ and where the Lamé constants in two dimensions become $\mu _\text {2D}= \kappa _S/3$ for pure shear deformations and $\lambda _\text {2D} = 2 \kappa _A/3$. The bulk modulus for pure area dilatations ($\boldsymbol{\epsilon } \propto \boldsymbol{1}$) reads $K_A = \mu _\text {2D} + \lambda _\text {2D} = (\kappa _S + 2 \kappa _A)/3$.

**Bending free energy** The bending or Helfrich free energy of the elastic sheet solely depends on the mean curvature, $1/R = (1/R_1 + 1/R_2) /2$, when the topology of the sheet stays the same [69]. Here, $R_1$ and $R_2$ are the principal radii of curvature, and the free energy reads,

$$\begin{aligned} \mathcal {F}_\text {b} = \frac{\kappa _b}{2} \int _{\mathcal {A}_\text {sh}} \Big ( \frac{1}{R} \Big )^2 \text {d}^2r_\text {sh} \,. \end{aligned}$$

where $\kappa _b$ is the bending constant. For a sheet discretized by a mesh of triangles, it can be shown that $\mathcal {F}_\text {b}$ depends on the relative orientation of neighboring triangles and can be rewritten as [70, 71]

8 $$\begin{aligned} \mathcal {F}_\text {b} = \frac{\sqrt{3}\kappa _b}{2} \sum _{\langle i,j \rangle } \theta _{ij} \,. \end{aligned}$$

Here, $\theta _{ij} = \arccos (\boldsymbol{n}_i \cdot \boldsymbol{n}_j)$ is the angle between the normal unit vectors $\boldsymbol{n}_i$, $\boldsymbol{n}_j$ of two neighboring triangles and the sum runs over all pairs of neighboring triangles. From this form of $\mathcal {F}_\text {b}$, the bending force $-\boldsymbol{\nabla }_{\boldsymbol{R}_\text {sh}} \mathcal {F}_\text {b}$ can again be calculated analytically as a function of $\boldsymbol{R}_\text {sh}$, as detailed in Ref. [68].

### Friction matrix

In this section we address the friction matrix $\boldsymbol{G}$ of Eq. (1) with its three contributions. This includes friction of the droplet motion due to viscous shear stresses in the fluid flow and due to the motion of the contact line, some phenomenological friction of the sheet to dampen its motion, as well as liquid–substrate friction between droplet and elastic sheet.

We first introduce the different contributions in Sects. 2.2.1–2.2.3 and then show the total friction matrix in Sect. 2.2.4.

#### Shear and contact-line friction

In Ref. [52] we explain in detail how shear and contact-line friction are derived, respectively, from the boundary integral equation and from an expression that relates viscous shear stresses to the contact-line motion. We do not repeat the derivation but mainly explain the final expressions. The friction matrix of the fluid droplet for the two contributions reads

9 $$\begin{aligned} \boldsymbol{G}_0^{(1)} = \boldsymbol{X}^{-1}(\boldsymbol{ C}_d + \boldsymbol{Y}) + \boldsymbol{P}_\text {cl}^\text {t}\boldsymbol{M} \boldsymbol{P}_\text {cl} \,. \end{aligned}$$

A further contribution $\boldsymbol{G}_0^{(2)}$ is due to liquid–substrate friction and addressed below.

The first term on the right-hand side of Eq. (9) derives directly from the boundary integral equation. The components of the three matrices assume the following values:

10 $$\begin{aligned} X_{ij}&= \int _{C_j} \boldsymbol{O}(\boldsymbol{r}_i - \boldsymbol{r}) \, \text {d}^2r / A_j \end{aligned}$$

11 $$\begin{aligned} Y_{ij}&=\int _{C_j} \boldsymbol{T}_\text {O}(\boldsymbol{r}_i - \boldsymbol{r}) \boldsymbol{n} \, \text {d}^2 r \, , \end{aligned}$$

where $\boldsymbol{O}$ is the Oseen tensor and $\boldsymbol{T}_\text {O}$ the associated stress tensor. The surface integral is performed over the polygonal cell $C_j$ with area $A_j$ constructed around mesh vertex *j* and $\boldsymbol{r}_i$ is the position of vertex *i*. For the explicit surface integration Gaussian quadrature is used, as detailed in Ref. [50]. The matrix $\boldsymbol{C}_d$ is diagonal with elements $c_i=1/2$, except if vertex *i* is on the contact line, then $c_i=\theta _i / 2\pi$ with contact angle $\theta _i$.

The second term on the right-hand side of Eq. (9) contains the friction matrix $\boldsymbol{M}$ of the contact line and follows from Moffatt’s early result [72] that relates the normal stresses along the liquid–gas interface to the contact-line velocity. After some integration [52], one obtains

12 $$\begin{aligned} M_{ij}= 2\eta \frac{A_i}{\zeta }\ln \left( \frac{\zeta }{l_\textrm{s}}\right) \frac{\sin ^2 \theta _i}{\theta _i - \sin \theta _i \cos \theta _i} \delta _{ij} \,, \end{aligned}$$

where $\theta _i$ is the contact angle at vertex *i*, $l_s$ is the liquid–substrate slip length, which we introduce further below, and ζ is a mesoscopic length, which in practice acts as a fitting parameter. Finally, $\boldsymbol{P}_\text {cl}$ is a projection operator that projects the vertex velocities of the contact line out of the droplet-mesh velocity vector $\dot{\boldsymbol{R}}_0$ but only takes the velocity components normal to the contact line and tangential to the substrate.

#### Elastic-sheet friction

For the friction of the elastic sheet, we introduce a phenomenological constant $\xi _\text {sh}$ mainly for computational reasons to dampen the dynamics of the elastic sheet and thereby ensure numerical stability, but also to capture dissipation inside the sheet and when there is contact to a second fluid. The chosen value assures that the sheet relaxes much faster than the droplet. Thus, we write

13 $$\begin{aligned} \boldsymbol{G}_\text {sh}^{(1)} = \xi _\text {sh} \boldsymbol{1} \,. \end{aligned}$$

An additional term $\boldsymbol{G}_\text {sh}^{(2)}$ is due to liquid–substrate friction, which we introduce now.

#### Liquid–substrate friction

The friction between substrate and the liquid droplet is governed by the Navier condition,

14 $$\begin{aligned} l_s \boldsymbol{P}_\text {t} \boldsymbol{\sigma }\boldsymbol{n} + \eta \boldsymbol{P}_\text {t} (\boldsymbol{v} - \boldsymbol{v}_\text {sh}) = 0 \,. \end{aligned}$$

It is a pure kinematic condition and sets the relative velocity $\boldsymbol{v} - \boldsymbol{v}_\text {sh}$ with which the droplet slips along the substrate. Here, $\boldsymbol{P}_\text {t} \boldsymbol{\sigma }\boldsymbol{n}$ is the viscous shear stress along the substrate, where $\boldsymbol{P}_\text {t} = \boldsymbol{1} - \boldsymbol{n} \otimes \boldsymbol{n}$ is the projector on the tangential plane normal to $\boldsymbol{n}$, thus any pressure term $-p \boldsymbol{1}$ in the stress tensor $\boldsymbol{\sigma }$ does not contribute. Finally, $l_s$ is the slip length and for $l_s \rightarrow 0$ one recovers the no-slip boundary condition.

Reference [52] demonstrates that the viscous shear stresses $\boldsymbol{\sigma }\boldsymbol{n} + p \boldsymbol{1}$ that all act on the droplet vertices are represented by $\boldsymbol{G}_0^{(1)} \dot{\boldsymbol{R}}_0$, where we introduced $\boldsymbol{G}_0^{(1)}$ in Eq. (9) as part of $\boldsymbol{G}_0$ in the dynamic equations (1). To implement the second term on the left-hand side of the Navier condition (14), we need to add a friction matrix $\boldsymbol{G}_0^{(2)}$ to $\boldsymbol{G}_0$ and define $\boldsymbol{G}_\text {slip}$. Thus we have

15 $$\begin{aligned} \boldsymbol{G}_0^{(2)} = \boldsymbol{P}_{\Vert 0}^\text {t} \boldsymbol{Q} \boldsymbol{P}_{\Vert 0} \hspace{5.0pt}\text {and} \hspace{5.0pt}\boldsymbol{G}_\text {slip} = - \boldsymbol{P}_{\Vert 0}^\text {t} \boldsymbol{Q} \boldsymbol{P}_{\Vert \text {sh}} \end{aligned}$$

with

16 $$\begin{aligned} Q_{ij} = \frac{\eta }{l_s} A_i \delta _{ij} \,, \end{aligned}$$

which only acts on the tangential velocity components at the liquid-sheet interface. The operators $\boldsymbol{P}_{\Vert 0}$, $\boldsymbol{P}_{\Vert \text {sh}}$ project these tangential velocities out of the respective velocity vectors $\dot{\boldsymbol{R}}_0$, $\dot{\boldsymbol{R}}_\text {sh}$. So, these operators are rectangular matrices. The transpose matrix $\boldsymbol{P}_{\Vert 0}^\text {t}$ in Eq. (15) then takes the resulting tangential vectors at the liquid-sheet interface and imbeds them into the higher dimensional vector space, where $\dot{\boldsymbol{R}}_0$ lives. With these additional friction matrices one fully satisfies the Navier condition since $- \boldsymbol{\nabla }_{\boldsymbol{R}_0} (\mathcal {F}_0 + \mathcal {F}_\text {cons})$ in Eq. (1) does not possess any tangential components besides at the contact line.

While the Navier condition is a kinematic constraint, we also have to balance tangential viscous shear stresses of the fluid with the in-plane elastic stresses in the sheet, $\boldsymbol{P}_\text {t} \boldsymbol{\sigma }\boldsymbol{n} = - \delta \mathcal {F}_\text {d}/\delta \boldsymbol{r}_\text {sh}$. So the Navier condition becomes $- \delta \mathcal {F}_\text {d}/\delta \boldsymbol{r}_\text {sh} = - \eta /l_s \boldsymbol{P}_\text {t} (\boldsymbol{v} - \boldsymbol{v}_\text {sh})$ and it is readily satisfied in Eq. (1) by adding

17 $$\begin{aligned} \boldsymbol{G}_\text {sh}^{(2)} = \boldsymbol{P}_{\Vert \text {sh}}^\text {t} \boldsymbol{Q} \boldsymbol{P}_{\Vert \text {sh}} \end{aligned}$$

to the elastic sheet friction matrix $\boldsymbol{G}_\text {sh}^{(1)}$ of Eq. (13).

#### Total friction matrix

Collecting all the contributions, we arrive at the following friction matrices that fill the total friction block matrix $\boldsymbol{G}$ in Eq. (1). For the full droplet friction matrix $\boldsymbol{G}_0 = \boldsymbol{G}_0^{(1)} + \boldsymbol{G}_0^{(2)}$ we have

18 $$\begin{aligned} \boldsymbol{G}_0 = \boldsymbol{X}^{-1}(\boldsymbol{ C}_d + \boldsymbol{Y}) + \boldsymbol{P}_\text {cl}^\text {t}\boldsymbol{M} \boldsymbol{P}_\text {cl} + \boldsymbol{P}_{\Vert 0}^\text {t} \boldsymbol{Q} \boldsymbol{P}_{\Vert 0} \,. \end{aligned}$$

The full elastic sheet friction matrix reads

19 $$\begin{aligned} \boldsymbol{G}_\text {sh} = \boldsymbol{G}_\text {sh}^{(1)} + \boldsymbol{G}_\text {sh}^{(2)} = \xi _\text {sh} \boldsymbol{1} + \boldsymbol{P}_{\Vert \text {sh}}^\text {t} \boldsymbol{Q} \boldsymbol{P}_{\Vert \text {sh}} \,. \end{aligned}$$

and the liquid-sheet friction matrix is

20 $$\begin{aligned} \boldsymbol{G}_\text {slip} = - \boldsymbol{P}_{\Vert 0}^\text {t} \boldsymbol{Q} \boldsymbol{P}_{\Vert \text {sh}} \,. \end{aligned}$$

### Final dynamic equations

For a complete solution of the dynamic equations (1), we still need to determine the Lagrange parameters $\lambda _i$ introduced in Sect. 2.1.2 and formalized in Eq. (5). We use here a method, which we employed in Ref. [50] to implement the volume constraint following Ref. [73], and generalize it to all our constraints. The idea is to move $- \boldsymbol{\nabla }_{\boldsymbol{R}} \mathcal {F}_\text {cons}$ in Eq. (1) to the left-hand side. For this we enhance the velocity vector $\dot{\boldsymbol{R}}$ by the column vector $\boldsymbol{\Lambda } = [\lambda _0, \lambda _1, \ldots , \lambda _N]$ and introduce the constraint matrix $\boldsymbol{G}_\text {cons} = [\boldsymbol{\nabla }_{\boldsymbol{R}} g_0(\boldsymbol{R}), \boldsymbol{\nabla }_{\boldsymbol{R}} g_1(\boldsymbol{R}), \ldots , \boldsymbol{\nabla }_{\boldsymbol{R}}$ $g_N(\boldsymbol{R})]$. Then, the enhanced system of dynamic equations reads,

21 $$\begin{aligned} \left[ \begin{array}{cc} \boldsymbol{G} &  \boldsymbol{G}_\text {cons} \\ \boldsymbol{G}_\text {cons}^\text {t}&  \boldsymbol{0} \end{array} \right] \left[ \begin{array}{c} \dot{\boldsymbol{R}} \\ \boldsymbol{\Lambda } \end{array} \right] = \left[ \begin{array}{c} - \boldsymbol{\nabla }_{\boldsymbol{R}} (\mathcal {F}_0 + \mathcal {F}_\text {sh}) \\ \boldsymbol{0} \end{array} \right] \,. \end{aligned}$$

While the upper row represents the original dynamic equations, the lower row states that for each constraint $\boldsymbol{\nabla }_{\boldsymbol{R}} g_i(\boldsymbol{R}) \cdot \dot{\boldsymbol{R}} = 0$, which indeed needs to be fulfilled. Thus, by inverting the enhanced friction matrix, we are able to calculate $\dot{\boldsymbol{R}}$ and thereby the dynamics of the combined droplet-sheet system.

We need to discuss a final point. The droplet and the sheet have their own meshes. However, for implementing the connectivity constraint at the droplet-sheet interface, the droplet and sheet vertices should coincide. To achieve this, we start with the position vectors $\boldsymbol{r}_{\text {sh}j}^{(I)}$ of the sheet vertices and determine the elastic forces acting on them, $-\boldsymbol{\nabla }_{\boldsymbol{r}_{\text {sh}j}^{(I)}} \mathcal {F}_\text {sh}$. Then, we perform a weighted interpolation to determine from these forces, the elastic force at the position $\boldsymbol{r}_{j}$ of a droplet vertex. The weighted interpolation is performed with the Julia program package Surrogates.jl. It uses the Inverse Distance Surrogate method and weighs the forces from sheet vertices surrounding $\boldsymbol{r}_{j}$ according to their inverse distance. After solving the dynamic equations (21), the sheet velocities $\dot{\boldsymbol{r}}_{j}$ are interpolated back on the velocities $\dot{\boldsymbol{r}}_{\text {sh}j}^{(I)}$ of the sheet vertices, which then perform a time step.

Finally, the dynamic equations (21) are discretized on a mesh of gridpoints, which we introduce in the following subsection. The discretizations of the elastic in-plane deformation and bending forces are explained in Sect. 2.1.3.

### Non-dimensionalization, parameters, and simulation details

**Non-dimensionalization** We non-dimensionalize the dynamic equations (1) as in Ref. [52]. We rescale lengths by the radius $R_0$ of the contact line on an initially plane substrate and energies by $\gamma _\text {lg} R_0^2$. To rescale time, we choose $\tau _0=9\gamma _\textrm{lg}^{-1}\eta R_0\ln (\zeta /l_\textrm{s})$, which results from the Cox–Voinov law for the velocity of the contact line and is an estimate for the time needed to move a distance $R_0$. Used in Eq. (1), the droplet properties only depend on the equilibrium contact angle $\theta _\text {eq}$ as well as the respective ratios $l_s/R_0$ and $\zeta /R_0$ for the slip length and the phenomenological length ζ. Since we rescale energies and time as mentioned, the shear viscosity η in non-dimensional units becomes $\tilde{\eta } = [9\ln (\zeta /l_\textrm{s})]^{-1}$.

**Droplet parameters** For the droplet parameters we follow Refs. [50, 52] and base them on a 90%-glycerol/10%-water mixture for which de Ruijter et al. [74] measured in experiments $\ln (\zeta /l_\textrm{s})=44$, $\eta =209\,\textrm{mPa}\,\textrm{s}$, $l_\textrm{s}=1\,\textrm{nm}$, $\gamma _\textrm{lg}=65.3\,\textrm{mN}\,\textrm{m}^{-1}$, and mass density $\rho =1.24\,\textrm{g}\,\textrm{ml}^{-1}$. In particular, to rescale the elastic constants of the sheet, we need $\gamma _\textrm{lg}$ and the length scale $R_0$, for which we set $R_0 = 100 \mu \text {m}$.

**Elastic-sheet parameters** The elastic moduli introduced in Sect. 2.1.3 for the thin elastic sheet can be linked to the elastic properties of the bulk elastic material. Typically, these properties are described by the Young modulus *E* that quantifies the relative elongation due to a tensile stress, $\sigma = E \Delta L/L$, and Poisson’s ratio that quantifies the relative lateral contraction, $\nu = - (\Delta d/ d) / (\Delta L/L )$. As an example, we take here an SIS (Styrol-Isopren-Styrol) elastomer used in Ref. [26] with $E = 1 \text {MPa}$ and ν=0.49, the latter indicating that the bulk material is nearly incompressible [75]. Furthermore, we can calculate the elastocapillary length, introduced in Sect. 2.1.1 as $l_\text {el} = \gamma _\text {lg} / E = 0.06 \mathrm {\mu m}$.

**Fig. 2:**
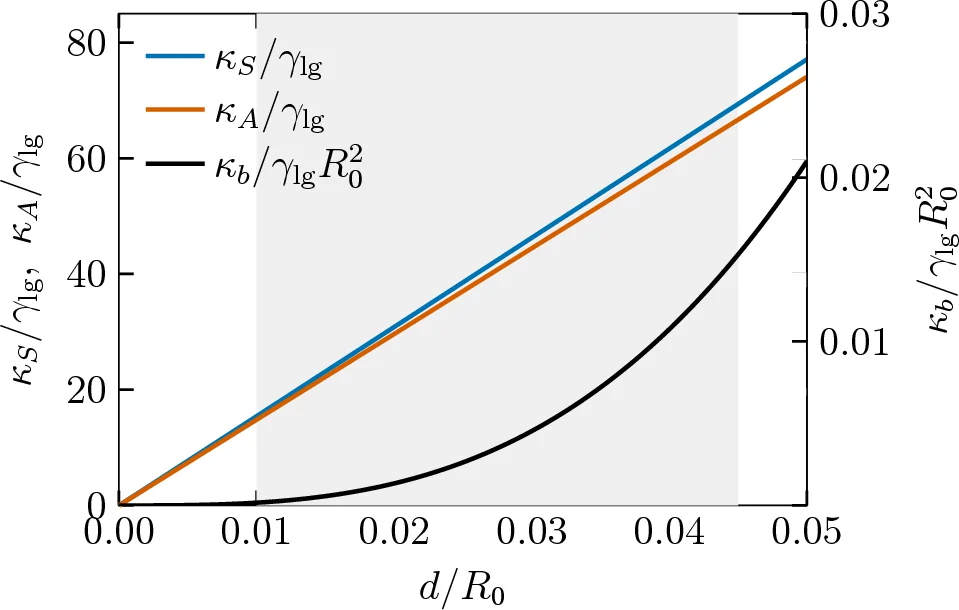
The shear ($\kappa _S$), area ($\kappa _A$), and bending ($\kappa _b$) elastic moduli plotted *vs.* film thickness *d*. The gray area indicates the thickness range used in our work

Shear, area, and bending moduli of a thin sheet of thickness *d* can be related to the bulk properties. Typically, one derives formulas within linear elasticity theory [76]. Matching them to the moduli of the Skalak and Helfrich free energies, we have

22 $$\begin{aligned} \kappa _S = \frac{3}{2} \frac{Ed}{1+\nu } \,, \,\, \kappa _A = \frac{3}{2} \frac{\nu E d}{1-\nu ^2} \,\, \text {and} \,\, \kappa _b = \frac{1}{12} \frac{ E d^3}{1-\nu ^2}\nonumber \\ \end{aligned}$$

In Fig. 2 we plot all three elastic moduli *versus* sheet thickness for the chosen parameters. We vary the sheet thickness in the range $d = 1 - 4.5 \mathrm {\mu m}$, which is indicated in the plot. While the two in-plane elastic moduli grow linearly in the sheet thickness, the bending modulus increases with $d^3$. So at small thicknesses, it becomes easier to bend the sheet compared to shearing it. In fact, at the end of Sect. 3.1 we will comment that the bending energy is always negligible compared to the in-plane deformation energy, in particular, for our largest thickness. This is also stated in literature [32].

Finally, we set the friction coefficient of the sheet in Eq. (13) to $\xi _{\text {sh}}=10^{-3} \, \gamma _{\text {lg}}\tau _0/R_0^2$, which results in a relaxation time much shorter than the characteristic time scale $\tau _0$ of droplet relaxation. Thus, the sheet performs a quasi-static dynamics.

**Initial condition** Regardless which equilibrium contact angle $\theta _\text {eq}$ we set, we always start the simulations with a droplet sitting on the plane sheet. The droplet begins with the shape of a spherical cap, a contact angle of $90^\circ$, and a contact line with radius $R_0$, which we use as a characteristic length to rescale all other lengths. This length is fixed by the droplet volume. Furthermore, the initially plane sheet is a square with a side length $4R_0$ and the droplet sitting in its center.

In our simulations we considered different cases. We either clamped all four edges of the sheet, meaning we fixed their positions throughout the simulation, or moved them apart gradually until a specific relative extension ϵ is realized (isotropic stress). We also apply a uniaxial stress with different conditions at the lateral edges. When we implement the relative extension ϵ, we let the droplet first relax during time t=0.05, which we identified as the typical relaxation time of the droplet toward the equilibrium configuration. Then, we pull the sheet edges apart always with the same speed until the relative extension ϵ is reached. This ensures that the droplet reaches the equilibrium state and does not exhibit numerical pinning.

**Meshes** All simulations are performed using triangle meshes, which are generated with the three-dimensional finite-element mesh generator gmsh. We employ coarser meshes for sheet and droplet in Sect. 3.1 and finer meshes with a resolution increased by a factor four in Sects. 3.2 and 3.3. The coarse mesh representing the droplet contains 307 vertices, 608 triangles, and the mean nearest-neighbor distance is $0.157 R_0$. The finer mesh is obtained by one refinement step in the mesh generator. It is made of 1219 vertices, 2432 triangles and the mean nearest-neighbor distance is $0.080 R_0$. The coarse mesh representing the sheet contains 513 vertices and 944 triangles, while the fine mesh is made of 1969 vertices and 3776 triangles.

**Fig. 3:**
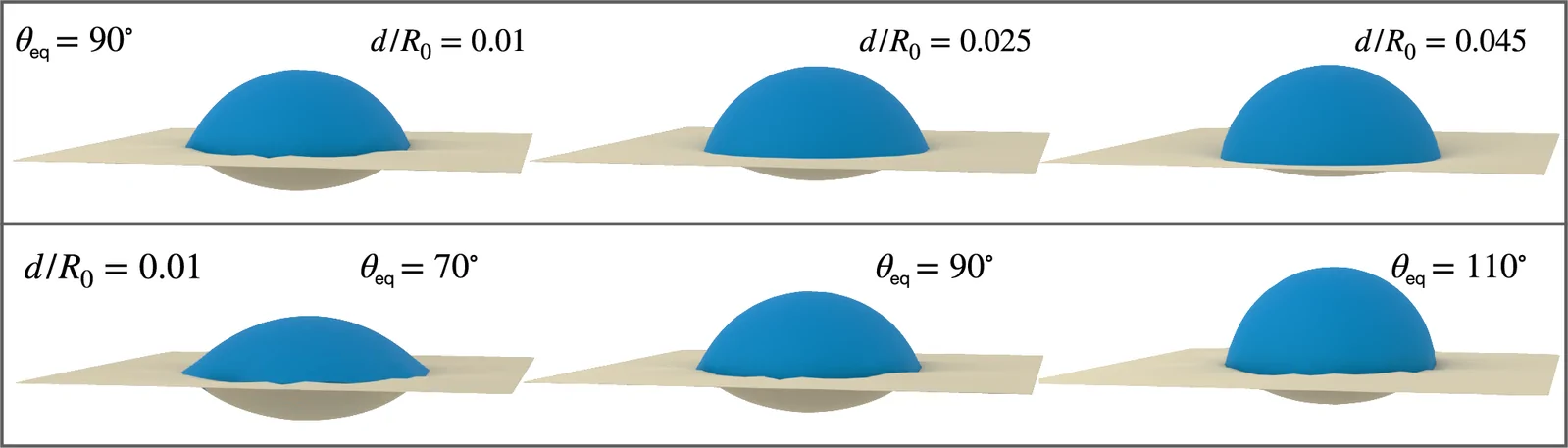
Droplets sitting on a thin elastic sheet. Upper row: Equilibrium contact angle $\theta _\text {eq} = 90^\circ$ and different values of sheet thickness $d/R_0$. Lower row: $d/R_0 = 0.01$ and different values of $\theta _\text {eq}$

## Results

We use the extended BEM to determine the equilibrium shape of a droplet that sits on an elastic sheet. The shape is determined by a number of control parameters and can be tuned via various protocols for the boundary conditions of the sheet. In Sect. 3.1 we clamped all edges of the elastic sheet and show how morphological metrics such as the height and the total macroscopic contact angle depend on intrinsic properties of the sheet, in particular, its thickness. In Sect. 3.2 we isotropically stretch the sheet and determine the radial profiles of azimuthal and radial stresses. Finally, in Sect. 3.3 we stretch the sheet along only one direction using different boundary conditions for the lateral edges and characterize the elongated shape of the droplet.

### Clamped elastic sheet

**Fig. 4:**
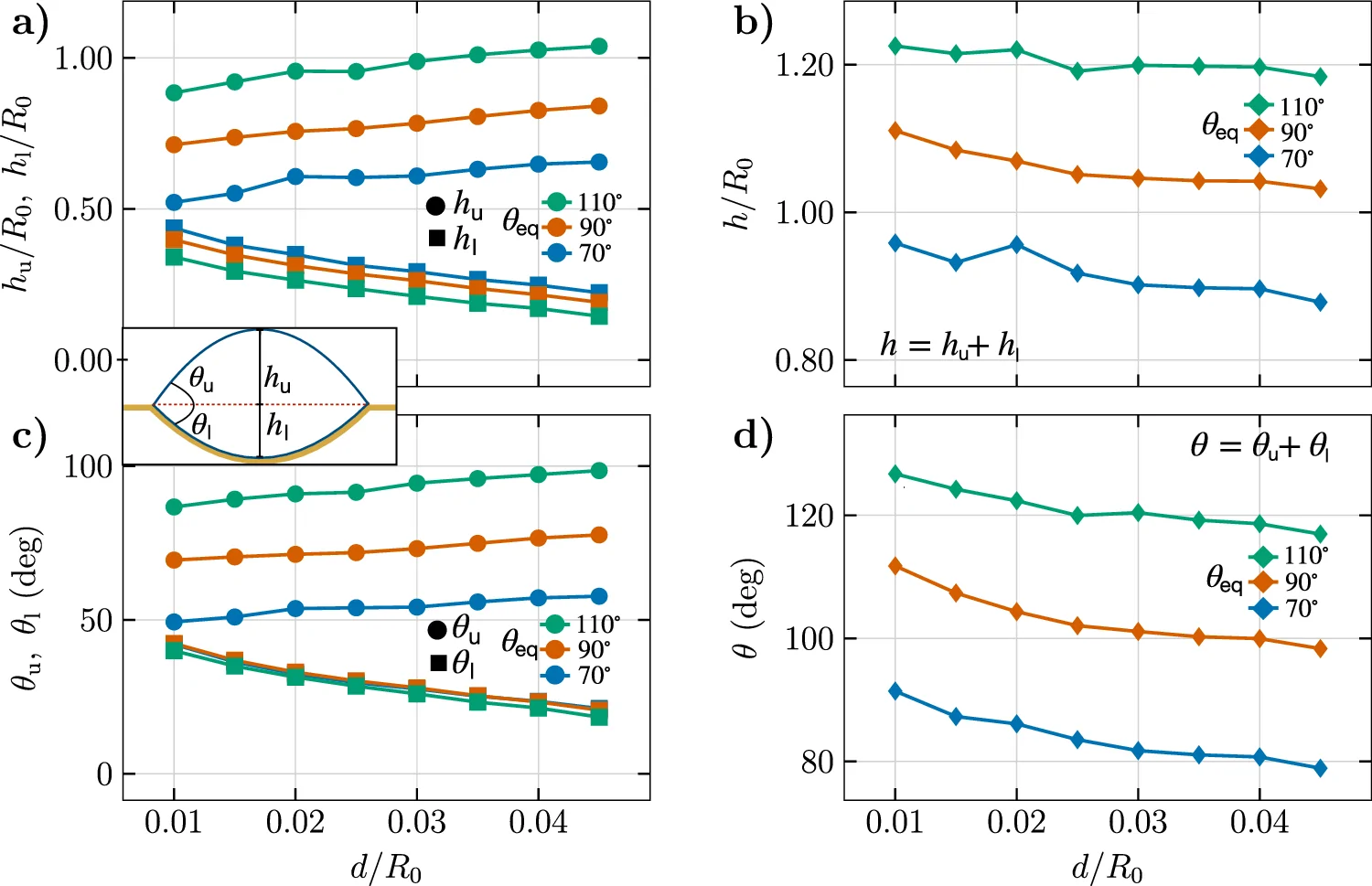
Morphological metrics of the droplet sitting on a sheet plotted versus sheet thickness $d/R_0$. **a** Height of lower ($h_\text {l}$) and upper ($h_\text {u}$) part of the droplet and **b** total height $h = h_\text {l} + h_\text {u}$ for several values of $\theta _\text {eq}$. **c** Macroscopic opening angle of lower ($\theta _\text {l}$) and upper ($\theta _\text {u}$) part and **d** total macroscopic contact angle $\theta = \theta _\text {l} + \theta _\text {u}$ for several values of $\theta _\text {eq}$

We clamped the four edges of the elastic sheet and studied in detail the resulting shape of the droplet sitting on the sheet for different equilibrium or Young’s angles $\theta _\text {eq}$ and sheet thicknesses *d*. The Laplace pressure in the droplet deforms the sheet and the droplet assumes a lens shape. The resulting equilibrium configurations in Fig. 3 give a first impression of the phenomenology. For increasing sheet thickness (first row), meaning increasing stiffness of the sheet, the droplet sinks less into the sheet and the lens shape becomes strongly asymmetric with respect to the horizontal plane defined by the contact line. Tuning the equilibrium angle (second row), the droplet sinks in more for smaller values of Young’s angle, because the contact area with the sheet is larger. For small thickness $d/R_0 = 0.01$, we observe small ripples around the contact line. They do not appear in the mesh of the sheet and, therefore, are an artifact when rendering the smooth surface from the mesh.

In Fig. 4 we study the droplet shape in more detail by presenting several morphological metrics, which we plot *versus* sheet thickness *d* for three values of Young’s angle. The contact line defines a plane, which we use to introduce the heights of the upper and lower part of the droplet [see inset between plots (a) and (c)]. Furthermore, assuming spherical caps for the two parts and fitting circle segments to vertical cross sections of the droplets, we define upper and lower opening angles [plot (c)]. Their sums define a macroscopic contact angle [77]. Plot (a) clearly demonstrates the observed phenomenology. For thicker sheets $h_l$ decreases and $h_u$ increases, while the total height $h=h_u + h_l$ [plot (b)] does not vary much. This is interesting, since the droplet transitions from a more symmetric to a highly non-symmetric shape with increasing thickness (see Fig. 3). A similar trend as for $h_l$ and $h_u$ is observed for $\theta _l$ and $\theta _u$, respectively [plot (c)].

Interestingly, the curves of $\theta _l$ for different $\theta _\text {eq}$ are almost identical; thus, independent of Young’s angle, and also the curves of $h_l$ do not vary much with $\theta _\text {eq}$. A possible reason is that the shape of the lower part of the droplet is strongly determined by the elastic sheet, while $\theta _\text {eq}$ influences both parts. Therefore, controlling $\theta _\text {eq}$, provides a method for selectively tuning the shape of the upper droplet half. Thereby, the optical properties of the droplet, used as a liquid lens, can be tuned, as we will discuss in Sect. 3.2.

The total macroscopic contact angle in plot (d) is always above $\theta _\text {eq}$. It does not agree with $\theta _\text {eq}$ due to the lens shape of the droplet, as we discuss in the next paragraph. Nonetheless, it should slowly converge toward $\theta _\text {eq}$ for large thicknesses, where the sheet is hardly deformed and the drop assumes the typical spherical-cap shape of a planar substrate. However, this limit is not realized at $d = 0.045 R_0$ as the non-zero values for $h_l$ show.

**Fig. 5:**
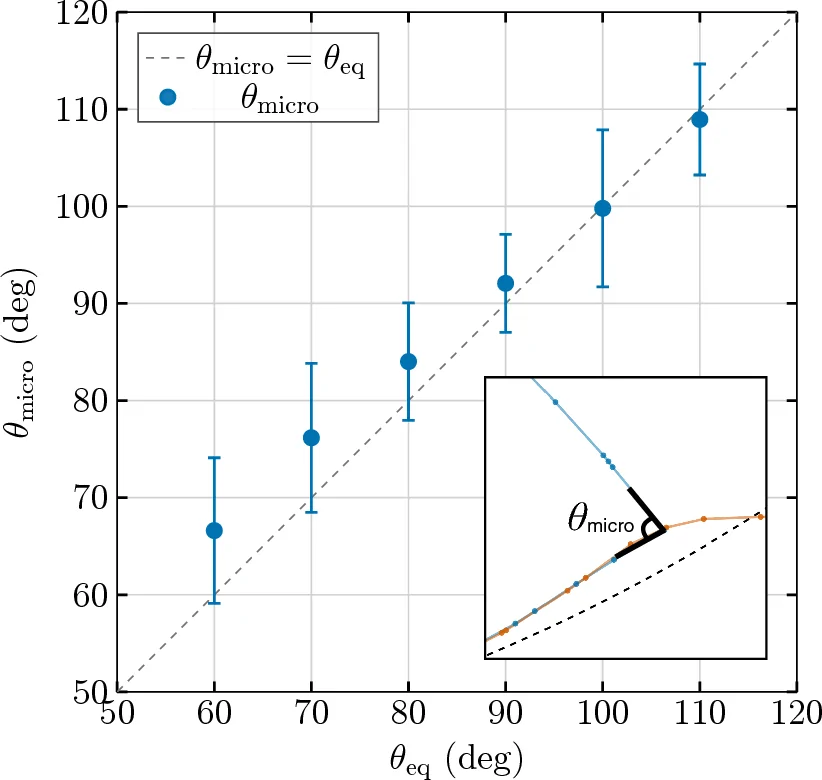
Microscopic contact angle plotted *vs* equilibrium contact angle for a sheet of thickness $d=0.02R_0$. The dashed line shows $\theta _\text {micro} = \theta _\text {eq}$. To determine $\theta _\text {micro}$ an average over 24 vertical cross sections of the droplet is taken, and the standard deviation is indicated. Inset: The angle $\theta _\text {micro}$ is determined from local tangents of the liquid–gas interface (blue) and sheet (brown) at the contact line. The dashed line shows the circular fit to the droplet-sheet interface extended to the region of the contact line

The deformation of the sheet close to the contact line is nicely illustrated in the inset of Fig. 5. The liquid–gas surface tension of the free droplet surface pulls at the contact line. The normal component is balanced by the normal bending stress due to the negative curvature of the sheet. The tangential sheet stresses at the contact line cancel each other, and the microscopic contact angle or Young’s angle should be realized. This is verified in the main plot of Fig. 5, where we plot the measured contact angle *versus* Young’s angle for sheets with thickness $d/R_0 = 0.02$. To generate the plot, we increased the linear mesh resolution by a factor two to better resolve the sheet deformation close to the contact line.

**Fig. 6:**
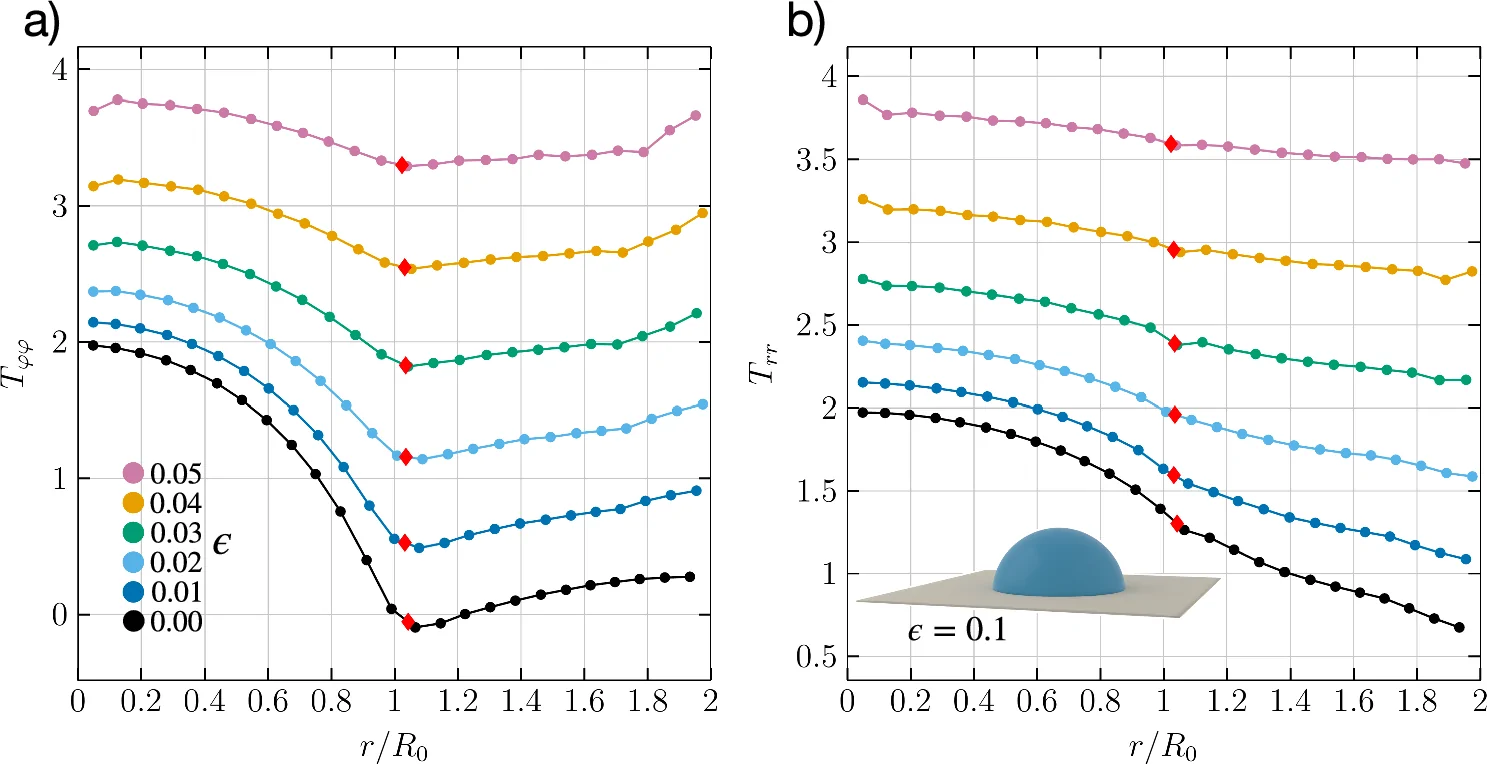
Azimuthal (**a**) and radial (**b**) stress components plotted *vs.* radial distance from the vertical axis for different relative extensions ϵ. Here, $T_{\varphi \varphi } = \boldsymbol{e}_\varphi \cdot \boldsymbol{T} \boldsymbol{e}_\varphi$, where $\boldsymbol{e}_\varphi$ is the azimuthal base vector, and $T_{rr} = \tilde{\boldsymbol{e}}_r \cdot \boldsymbol{T} \, \tilde{\boldsymbol{e}}_r$, where $\tilde{\boldsymbol{e}}_r$ is tangential to the sheet pointing radially outward. Both stress components are determined from an average over the azimuthal direction. The red diamonds indicate the location of the contact line. Inset in **b**: At an extension ϵ=0.1 (stress components are not shown), the sheet is roughly planar. Other parameters are sheet thickness $d=0.02 R_0$ and $\theta _\text {eq} = 90^\circ$

We add two final notes about the elasticity regime. First, from Fig. 4(a) we realize that the height $h_l$ of the lower part of the droplet assumes values up to $h_l=0.5 R_0$. Assuming that the radius of the circular contact line remains roughly the same, the area of the lower spherical cap is $\pi (R_0^2 + h_l^2) = 1.25 \pi R_0^2$. So the increase in area compared to the flat sheet-droplet interface before the deformation is 25%, which corresponds to a linear increase in one direction by 12%. This is at the upper limit of the validity of the linear elasticity regime. Second, for the same droplet geometry, if we compare bending and an estimate of the area dilatation energy using the moduli in Fig. 2 at our largest thickness, we realize that bending energy is always negligible compared to the in-plane deformation energy.

### Isotropically stretched sheet

We also studied droplets on elastic sheets, which we stretch in both directions up to a defined relative extension ϵ. Because of the increasing tension within the sheet, the lens-shaped droplet is “lifted up” until the sheet is flat again and the droplet assumes its equilibrium shape for a plane substrate. In the inset of Fig. 6(b) we illustrate this situation for ϵ=0.1 with the equilibrium angle $\theta _\text {eq} = 90^\circ$. The attached video in the supplemental material shows how the droplet first sinks into the sheet, which then is slowly stretched up to ϵ=0.1. The main plots (a) and (b) in Fig. 6 show the relevant stress components as a function of radial distance *r* from the vertical through the center of the droplet for different relative extensions ϵ up to 0.05. To calculate the stress components, we rely on the Cauchy stress tensor of the sheet given in spatial (Eulerian) coordinates:

23 $$\begin{aligned} \boldsymbol{T} = \frac{2}{\Delta } \, \boldsymbol{F} \, \frac{\partial f_\text {d}}{\partial \boldsymbol{C}} \, \boldsymbol{F}^\text {t} \,. \end{aligned}$$

Here, $2 \partial f_\text {d} / \partial \boldsymbol{C}$ is the second Piola–Kirchhoff stress tensor, which derives from the Skalak free energy density in Eq. (6) in material coordinates. The deformation gradient tensor $\boldsymbol{F}$ transforms it to spatial coordinates and $\Delta = \Vert \boldsymbol{F}_1 \times \boldsymbol{F}_2 \Vert$ is the two-dimensional determinant to account for changes in local area in the deformed sheet. It is written as cross product of vectors $\boldsymbol{F}_\alpha$ with components $F_{i\alpha }$ from the deformation gradient tensor. We take the stress tensor $\boldsymbol{T}$ and determine the diagonal components along the azimuthal ($T_{\varphi \varphi }$) and radial ($T_{rr}$) directions, where radial here means in the plane of the sheet. We plot the stress components, averaged over the azimuthal angle φ, in Fig. 6(a) and (b), respectively.

In general, the stresses increase with extension ϵ and the variation along *r* is largest for smallest ϵ. Here, the droplet causes the largest deformation of the sheet. The contact line (red diamonds) is roughly situated close to an inflection point, where the curvature in the stress curves changes sign. While for small ϵ the droplet is clearly recognizable in both stress curves, the radial component $T_{rr}$ becomes nearly linear in *r* with increasing ϵ. We observe that at large ϵ close to r=0 the stress curve is not horizontal as required by cylindrical symmetry, which is due to the fact that only a small number of stress values are available for performing an average along the azimuthal direction in order to calculate $T_{\varphi \varphi }$ and $T_{rr}$.

**Fig. 7:**
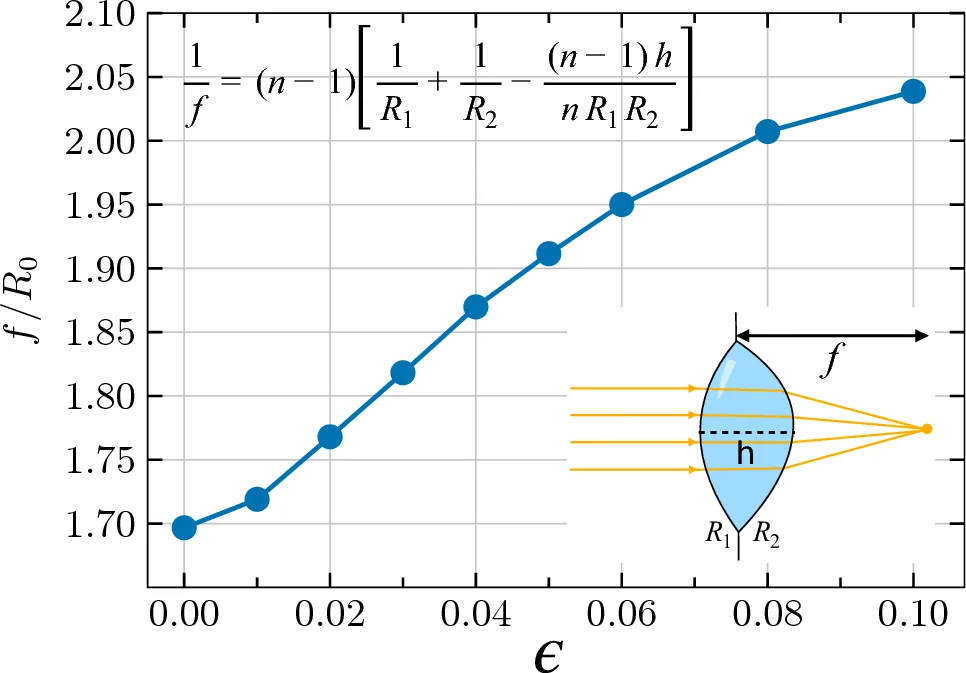
Focal length *f* of the lens-shaped droplet plotted versus the relative extension ϵ. Lower inset: Illustration of the focal length; *h* is the thickness of the droplet and $R_1$, $R_2$ are the two curvature radii of the two droplet halves (see Appendix A). Upper inset: The lens maker’s equation of the focal length. The refractive index n=1.46 of the 90%-glycerol/10%-water mixture is used [78]

Also, close to r=2 the component $T_{\varphi \varphi }$ bends upwards more strongly; this is at the edge of the sheet, where the cylindrical symmetry is no longer valid. Finally, for ϵ=0 there is a small region close to the contact line, where $T_{\varphi \varphi }$ becomes negative. Such compressional stresses are necessary to observe wrinkling in the sheet [23]. However, in our case these stresses seem to be too small since we do not observe any wrinkling.

Liquid lenses offer the ability to change their optical properties very rapidly in contrast to conventional lens systems and have already reached commercial use [41]. Now, our study of lens-shaped droplets enable us to have full access to the optical properties of the lens through its shape and, most importantly, to tune the optical properties by applying tension to the elastic sheet. The focal length (see lower inset of Fig. 7) is the most important quantity of a lens since it determines the magnification and angle of view. We used the lens maker’s equation (see upper inset of Fig. 7) to calculate the focal length for the lens-shaped droplet as a function of the relative extension (see Fig. 7). Applying tension offers the possibility to tune the focal length, dynamically and reversibly, by about 15%. Interestingly, this range agrees roughly with the variation of the main lens in a typical smartphone. The curve in Fig. 7 has an S shape and tends toward a constant value for increasing ϵ that corresponds to the spherical-cap shape on a plane substrate.

### Uniaxially stretched sheet

**Fig. 8:**
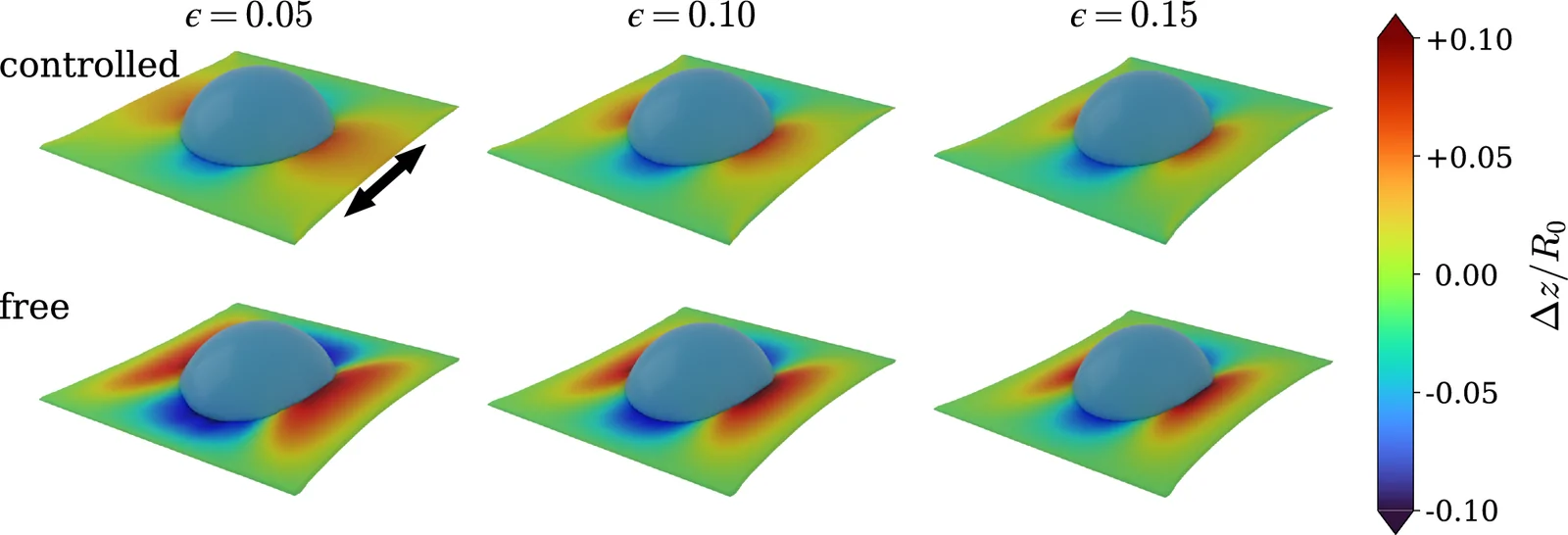
Droplets sitting on sheets stretched in one direction, as indicated by the double arrow, for controlled (top) and free (bottom) boundary conditions of the lateral edges. The columns correspond to three relative extensions ϵ. The heatmap rendering of the sheet shows the vertical displacement Δz relative to the initially plane sheet

Experiments show that droplets acquire an elongated shape if a uniaxial stress is applied to the sheet [27, 28]. To study this setting, we stretch the elastic sheet along the *x* direction with a relative extension ϵ, while for the lateral edges we choose three different boundary conditions. First, we clamp the lateral edges so that they do not move. Second, in what we refer to as the controlled boundary condition, we let them move inwards by the relative extension $\epsilon _y = - \nu \epsilon$, where ν=0.49 is the Poisson ratio of the bulk material. Thus, $\epsilon _y$ is chosen just like it would occur in a uniaxially stretched sheet without a droplet. Third, the lateral edges can move freely.

For the controlled and free boundary conditions, Fig. 8 shows droplets sitting on uniaxially stretched sheets for three values of ϵ. The elongation of the droplets along the stretching direction is clearly visible, in particular for the case of the free lateral edges (bottom). The heatmap on the sheet shows the vertical displacement relative to the plane sheet at the start of the simulation. Between the free edges and the droplet (bottom row) the sheet folds upward since the edges move inward, while in the stretching direction dimples occur. This shows that the contact line is not planar; it is elevated along the lateral direction and lowered along the stretching direction. Furthermore, the amount of vertical displacement decreases for increasing ϵ, meaning when stresses are larger. The same qualitative behavior can be observed for controlled lateral edges (top row) but the vertical displacements are smaller compared to the free edges. The latter have larger inward lateral displacements and, therefore, the folds are more pronounced.

**Fig. 9:**
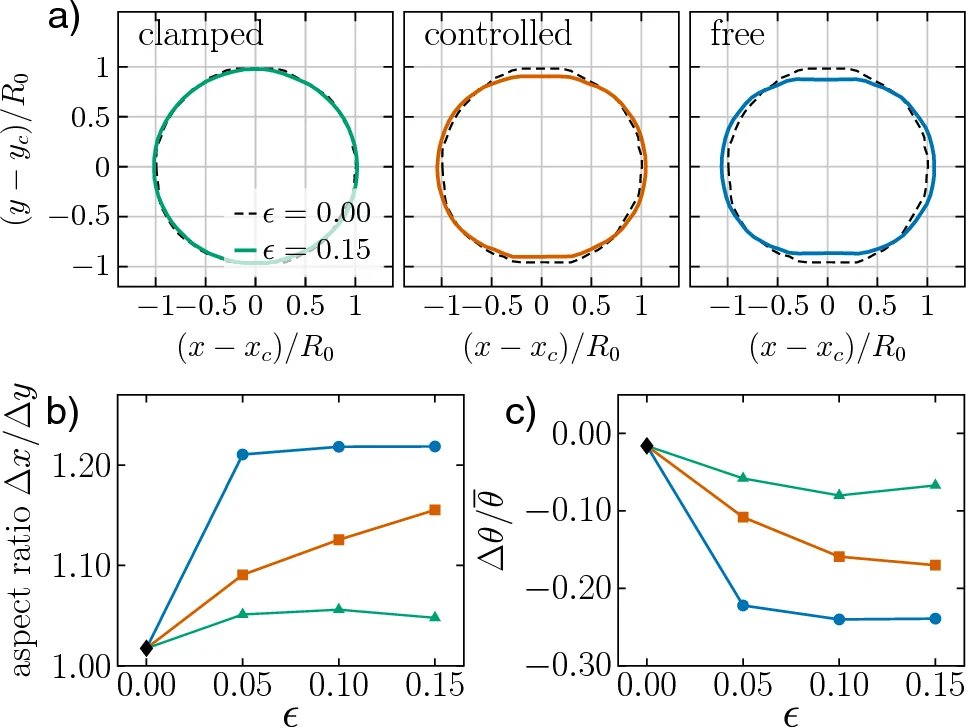
(**a**) Footprints of the contact line of a uniaxially stretched sheet with extension ϵ=0.15 for the three boundary conditions with clamped, controlled, and free lateral edges. The dashed line is the reference case for the clamped sheet with ϵ=0. (**b**) Aspect ratio of the contact line, $\Delta x / \Delta y$, and (**c**) the relative anisotropy of the total macroscopic contact angle, $\Delta \theta / \overline{\theta }$, plotted as a function of ϵ for the three boundary conditions. The same color code as in (**a**) is chosen

In Fig. 9 we present a quantitative analysis of the elongated droplet for the three boundary conditions. For an extension ϵ=0.15, Fig. 9(a) shows the footprint of the contact line, which is formed by projecting the contact line on the horizontal plane. The dashed line is the contact line of the unstretched sheet. Again, for the free lateral edges (blue, on the right) the elongation of the droplet along the stretching direction is largest, and for the sheet with clamped lateral edges (green, on the left) the elongation is the smallest. In Fig. 9(b) the aspect ratio $\Delta x/ \Delta y$ of the major axis to the minor axis of the footprint is plotted *versus* ϵ for all three boundary conditions. The ratio is close to one for the clamped case (below 1.06) and even decreases beyond ε=0.1. This behavior is expected since for large stresses the sheet becomes planar again, which means the droplet recovers a circular contact line. Interestingly, the aspect ratio in the case of free lateral edges is roughly constant for the non-zero values of ϵ although the vertical displacements of the sheet do indeed vary. For the controlled lateral edges the aspect ratio increases with ϵ throughout the entire range of simulated values of ϵ. One major finding of the whole study is that elongation of the droplet along the stretching direction is due to the droplet experiencing different sheet stiffnesses along the stretching and the lateral directions, which then determines the elongated shape of the droplet when sinking into the sheet. However, for protocols which do clamp the lateral edges, the sheet becomes flat again under large extensions and the droplet assumes its spherical-cap shape. Interestingly, the observed aspect ratios are similar to the values reported in Ref. [27].

The anisotropy of the droplet shape is also visible in the total macroscopic contact angles along the stretching ($\theta _x$) and lateral ($\theta _y$) directions. We measure these angles by fitting circles to the upper and lower droplet halves as before, but now in both directions. In Fig. 9(c) we plot the relative anisotropy

24 $$\begin{aligned} \frac{\Delta \theta }{\overline{\theta }} = \frac{\theta _{x} - \theta _{y}}{(\theta _{x} + \theta _{y})/2} \end{aligned}$$

*versus* ϵ. The anisotropy is negative indicating that along the stretching direction the droplet shape runs more flat and in agreement with the positive aspect ratio of the contact line. The magnitude of the anisotropy is smallest for the clamped case and largest for the free lateral edges, where the anisotropy again is nearly constant for the non-zero values of ϵ.

## Conclusions

In this article we extended our formalized BEM [52], which we developed earlier to treat the dynamics of droplets on an undulating substrate. The BEM includes an elastic sheet as a substrate and determines the dynamics of the deforming sheet induced by the wetting droplet. Thus, in our formalized BEM the total friction matrix is extended by contributions for the elastic sheet and coupling terms between sheet and droplet that derive from the Navier slip condition and a force balance at the droplet-sheet interface. The free energy of the total system now includes the Skalak and Helfrich free energies for in-plane and bending deformations of the sheet, respectively. Real elastic parameters of an elastomer are chosen to calculate the shear, area, and bending moduli as a function of the sheet thickness.

To test our method, we studied three specific protocols. First, we thoroughly characterized the lens shape of the droplet sitting on a clamped elastic sheet using morphological metrics. They include the heights and macroscopic opening angles of the two droplet halves, which determine the total height and macroscopic contact angle. Interestingly, the response of the lower droplet half to variations of the sheet thickness is nearly independent of Young’s angle. This offers the possibility to selectively tune the shape of the upper droplet part while keeping the lower droplet half unchanged. Therefore, our finding may open avenues for enhanced control of liquid optical lenses. Finally, zooming into the region around the contact line, the microscopic contact angle agrees with Young’s angle, as expected.

Second, we stretched the sheet in all four directions equally. The droplet is “lifted up” until the sheet becomes flat again and assumes its spherical-cap shape, typical for planar substrates. Using the stress tensor derived from the Skalak free energy, we are able to extract the radial profiles of azimuthal and radial elastic stresses for several sheet extensions ϵ from our simulation data. Droplets on elastic sheets take the form of liquid lenses, which are already used in commercial applications. The droplet shape, changing with ϵ, manifests itself in an increasing focal length. Thus, applying tension to the elastic sheet offers the possibility to tune the optical properties of such liquid optical lenses.

From a more fundamental perspective, the droplet creates a deformation field in the elastic sheet. Similar to capillary interactions, which manifest themselves in the Cheerios effect [79], the sheet will mediate elastocapillary interactions between two droplets, which in the simplest setting should be attractive. Controlling the tension in the sheet thus offers the possibility of tuning these interactions and, thereby, the assembly of droplets.

Finally, we stretched the sheet along one direction using different boundary conditions for the lateral edges. Due to the coupling between sheet and droplet, the shape of the droplet becomes elongated, the vertical sheet displacement shows upward folds and dimples, and the contact line is no longer planar. We thoroughly characterized the droplet shape by the aspect ratio of the footprint of the contact line and the anisotropy in the macroscopic contact angle.

The reported work using the extended BEM opens several exciting avenues for future research. We plan to address wrinkling patterns around droplets, which we already see when we leave all edges of the sheet free. In a gradient of tension within the sheet, we expect the droplet to perform durotaxis. The possibility to study elastocapillary interactions between lens-shaped droplets and to develop an effective interaction potential is also very appealing. Under a uniaxial tension the effective potential for droplets with an elongated shape will be anisotropic and might cause novel complex dynamics. Finally, droplets sitting on an elastic sheet can be used as liquid optical lenses. Owing to their highly and easily tunable focal length, these lenses fill a market niche left open by conventional optical instruments. Thus, our work also opens novel avenues for tuning the focal length of liquid lenses by controlling the tension applied to the sheet.

## Supplementary Information

Below is the link to the electronic supplementary material.

Supplementary file 1 (mp4 2582 KB)

## Figures and Tables

**Fig. 10:**
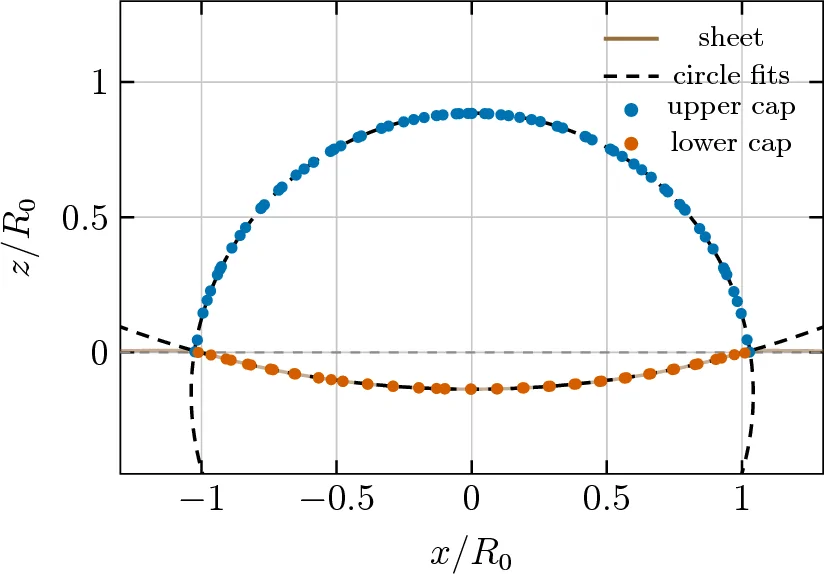
Cross section of the lens-shaped droplet sitting on an elastic sheet under isotropic extension with ϵ=0.05 and thickness d=0.02 $R_0$. Dots mark the points at which the droplet surface mesh intersects the vertical plane. Blue dots represent the droplet’s free surface in contact with the surrounding air, and orange dots belong to the droplet surface in contact with the sheet. Dashed lines show circular arcs fitted to the dots of same color

## Data Availability

The datasets generated and/or analyzed during the current study are available on reasonable request.

## Appendix A. Curvature radii of the two droplet halves

To determine the focal length of the lens-shaped droplet in Fig. 7, we model the two droplet halves by spherical caps (see Fig. 10). While this is expected for the upper droplet half due to the uniform hydrostatic pressure within the droplet, the spherical cap is a very good approximation also for the lower droplet surface in contact with the elastic sheet. The circular fit in Fig. 10 demonstrates the approximation for a representative case. As discussed in connection with Fig. 5, only very close to the contact line the droplet deviates from the spherical-cap shape.
